# Occupational noise and hypertension in Southern Chinese workers: a large occupational population-based study

**DOI:** 10.1186/s12889-024-18040-9

**Published:** 2024-02-21

**Authors:** Shanyu Zhou, Shijie Hu, Kexin Ding, Xianzhong Wen, Xudong Li, Yongshun Huang, Jiabin Chen, Dafang Chen

**Affiliations:** 1 Department of Epidemiology and Biostatistics, School of Public Health, Peking University, 100191 Beijing, China; 2 Guangdong Province Hospital for Occupational Disease Prevention and Treatment, 510300 Guangzhou, Guangdong China

**Keywords:** Occupational noise, Bilateral high-frequency hearing threshold on average, Bilateral high-frequency average hearing loss, Hypertension, Blood pressure

## Abstract

**Introduction:**

An increasing number of original studies suggested that occupational noise exposure might be associated with the risk of hypertension, but the results remain inconsistent and inconclusive. In addition, the attributable fraction (AF) of occupational noise exposure has not been well quantified. We aimed to conduct a large-scale occupational population-based study to comprehensively investigate the relationship between occupational noise exposure and blood pressure and different hypertension subtypes and to estimate the AF for hypertension burden attributable to occupational noise exposure.

**Methods:**

A total of 715,135 workers aged 18–60 years were included in this study based on the Key Occupational Diseases Surveillance Project of Guangdong in 2020. Multiple linear regression was performed to explore the relationships of occupational noise exposure status, the combination of occupational noise exposure and binaural high frequency threshold on average (BHFTA) with systolic and diastolic blood pressure (SBP, DBP). Multivariable logistic regression was used to examine the relationshipassociation between occupational noise exposure status, occupational noise exposure combined with BHFTA and hypertension. Furthermore, the attributable risk (AR) was calculated to estimate the hypertension burden attributed to occupational exposure to noise.

**Results:**

The prevalence of hypertension among occupational noise-exposed participants was 13·7%. SBP and DBP were both significantly associated with the occupational noise exposure status and classification of occupational noise exposure combined with BHFTA in the crude and adjusted models (all *P* < 0·0001). Compared with workers without occupational noise exposure, the risk of hypertension was 50% greater among those exposed to occupational noise in the adjusted model (95% CI 1·42–1·58). For participants of occupational noise exposed with BHFTA normal, and occupational noise exposed with BHFTA elevated, the corresponding risks of hypertension were 48% (1·41–1·56) and 56% (1·46–1·63) greater than those of occupational noise non-exposed with BHFTA normal, respectively. A similar association was found in isolated systolic hypertension (ISH) and prehypertension. Subgroup analysis by sex and age showed that the positive associations between occupational noise exposure and hypertension remained statistically significant across all subgroups (all *P* < 0.001). Significant interactions between occupational noise status, classification of occupational noise exposure combined with BHFTA, and age in relation to hypertension risk were identified (all *P* for interaction < 0.001). The associations of occupational noise status, classification of occupational noise exposure combined with BHFTA and hypertension were most pronounced in the 18–29 age groups. The AR% of occupational noise exposure for hypertension was 28·05% in the final adjusted model.

**Conclusions:**

Occupational noise exposure was positively associated with blood pressure levels and the prevalence of hypertension, ISH, and prehypertension in a large occupational population-based study. A significantly increased risk of hypertension was found even in individuals with normal BHFTA exposed to occupational noise, with a further elevated risk observed in those with elevated BHFTA. Our findings provide epidemiological evidence for key groups associated with occupational noise exposure and hypertension, and more than one-fourth of hypertension cases would have been prevented by avoiding occupational noise exposure.

**Supplementary Information:**

The online version contains supplementary material available at 10.1186/s12889-024-18040-9.

## Introduction

Occupational noise is the exposure at the workplace to unpleasant or unwanted sounds [1], common in manufacturing, mining, construction, building materials, and other pillar industries in the national economy. According to the WHO/ILO, the pooled prevalence of occupational exposure to noise (≥ 85 dBA) among the general population of workers was 0·17 (95% CI: 0·16 − 0·19) [2]. An estimated 32·6 million workers have been exposed to occupational noise in China [3], of which 24·6% were in Guangdong. Noise-induced hearing loss is the most commonly recognized occupational disease in industrialized countries [4]. In addition to the traditional auditory effects, more attention has been given to the non-auditory health effects of occupational noise exposure, such as cardiometabolic system diseases, especially hypertension [5]. However, compared with the well-established relationship between occupational noise exposure and hearing loss [4, 6–8], the non-auditory health effects of occupational noise exposure on hypertension are controversial. Eight systematic reviews conducted in the last 20 years highlight occupational noise as a risk factor contributing to hypertension. Pooled risk estimates from systematic reviews for the development of arterial hypertension of occupational noise-exposed employees were reported to range from 1·07 to 2·56 [9–16]. However, the studies included in systematic reviews are highly heterogeneous, which may have influenced pooled estimates. Moreover, some cohort studies found no increased risk of hypertension associated with occupational noise exposure [17–19], challenging the role of occupational noise exposure as an established risk factor for hypertension.

Although there are many epidemiologic studies on the effects of occupational noise exposure on hypertension, the results are inconsistent, and their quality varies. Many studies lack appropriate non-exposed controls, have small sample sizes, and do not adjust for co-exposure factors. Moreover, the majority of previous studies were conducted with the general population in national databases and with the occupational population in cities or factories. In addition, most studies were carried out to explore the association of occupational noise exposure with hypertension, and few studies have been performed to comprehensively investigate the relationship between occupational noise exposure and blood pressure and different hypertension subtypes simultaneously. Furthermore, occupational noise exposure assessed by historical measurement records of working site could potentially result in underestimations of the true adverse health effects, as the use of hearing protective devices which may substantially reduce actual personal exposure to loud noise in the workplace. Bilateral high-frequency average hearing loss (BHFHL) is associated with cumulative occupational noise exposure, and Binaural high frequency (3, 4, and 6 kHz) threshold on average (BHFTA) level can serve as an objective biomarker for actual personal exposure to occupational noise [20–23]. However, the association between occupational noise combined BHFHL has rarely been evaluated among different hypertension classifications, and existing evidence is limited.

China is the country with the largest labor force in the world. There were 750·6 million employed people in China, accounting for 53·2% of the total population [24]. Given the high prevalence of occupational noise exposure in China, it is crucial to comprehensively identify the work-related associations between occupational noise exposure and hypertension in Chinese populations. Therefore, we conducted a large-scale occupational population-based study to investigate the association between occupational noise exposure, combined with BHFTA and hypertension subtypes using data from the key occupational disease surveillance project in Guangdong, China.

## Methods

### Study design and data source

We conducted a large-scale cross-sectional study to investigate the relationship between occupational noise exposure and hypertension using data from Guangdong’s Key Occupational Diseases Surveillance Project in 2020. The Key Occupational Diseases Surveillance Project of Guangdong was a provincial project to assess the health status of the occupational population exposed to noise, dust, benzene, Pb, and high temperature and to address the ever-increasing challenges of occupational diseases. The project has set up 122 surveillance units covering all counties of 21 cities in Guangdong, which is 100% coverage of counties and cities. There are 178 certified occupational health checkup organizations that undertake the occupational health examination of workers exposed to occupational hazards. Data from occupational health examinations were collected by trained health physicians and uploaded to the “Guangdong Internet Plus Occupational Disease Prevention and Occupational Health Management System”. Regular quality controls were conducted in occupational health checkup organizations, key occupational diseases surveillance departments at the county and city levels to verify the completeness of reporting. All data are centralized at the provincial key occupational diseases surveillance departments to make a final quality control, and quality assurance is further ensured by provincial key occupational diseases surveillance experts who randomly visit surveillance sites.

### Procedures

**Fig. 1:**
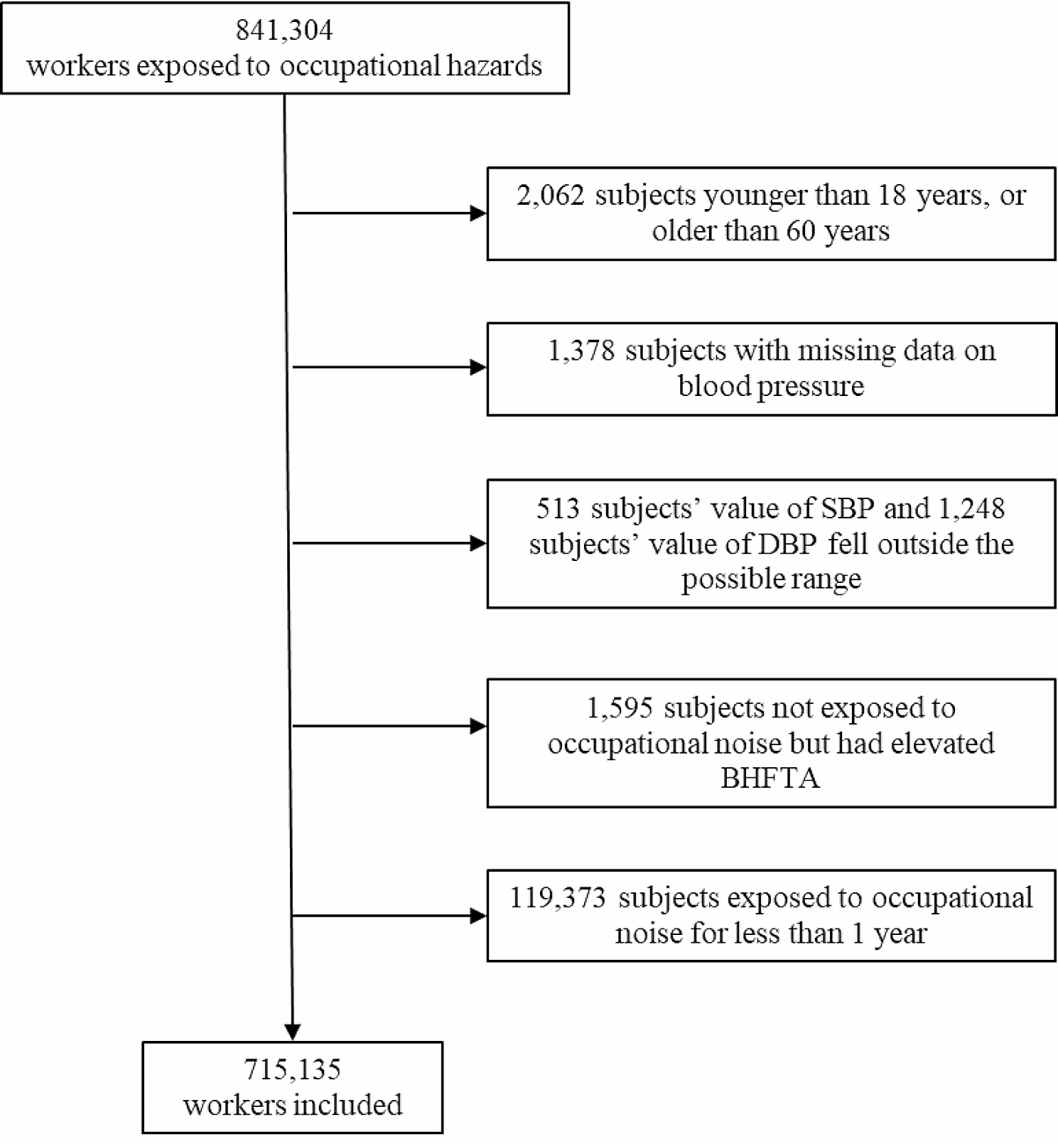
Flow diagram of workers exposed to occupational hazards and attending occupational health checkups with pure tone audiometry (PTA) test in Guangdong and were included in the present analysis

All data were extracted directly from the occupational health surveillance system of Guangdong. A total of 841,304 workers exposed to occupational hazards and attending occupational health checkups with pure tone audiometry (PTA) test from Jan 1, 2020, to Dec 31, 2020, in Guangdong were studied. We excluded subjects younger than 18 years, or older than 60 years (*n* = 2,062), subjects with missing data on blood pressure (*n* = 1,378); subjects’ value of systolic blood pressure (SBP) and diastolic blood pressure (DBP) fell outside the possible range (SBP: *n* = 513, DBP: *n* = 1,248), and those who were not exposed to occupational noise but BHFTA elevated (*n* = 1,595) because their BHFHL may have been caused by reasons other than occupational noise exposure; and those who were exposed to occupational noise for less than 1 year (*n* = 119,373) because their hypertension may not have been related to occupational noise exposure, attenuating causal bias. Finally, 715,135 workers who met the criteria were recruited (Fig. 1).

In this study, we used basic information, pure tone audiometry tests, and blood pressure. The basic information consisted of two sections: (1) Personal information, including name, sex, date of birth, exposure to occupational hazards, and years of exposure to occupational hazards. (2) Company information, including company name, address, and classification of industry. We additionally checked and excluded missing and incorrect values in all variables.

### Classification of exposure to occupational hazards

In our study, occupational hazard exposure included occupational noise exposure, dust exposure, benzene exposure, lead exposure, and high temperature exposure. Occupational noise exposure was defined as working in the presence of sound harmful to health, with an equivalent sound level of at least 80 dB(A) during an 8-hour workday or 40-hour workweek. Occupational dust exposure was defined as working in the presence of industrial dust, including inorganic dust, organic dust, and mixed dust. Occupational high-temperature exposure was defined as working in sites where the WGBT index was ≥ 25 °C. Occupational Pb exposure was defined as working in the presence of Pb dust or Pb smoke. Occupational benzene exposure was defined as working in the presence of benzene.

Information on occupational exposure, including name of occupational hazards and duration of exposure, was obtained via a questionnaire completed by the participants’ company according to the occupational health monitoring reports conducted by certified occupational health inspection organizations. We divided exposure to occupational hazards into non-exposed and exposed.

“Years of occupational hazard exposure” denotes the cumulative number of years an individual has been exposed to any one or a combination of occupational hazards, which includes noise, benzene, dust, Pb, and high temperatures, among others. For the noise non-exposed group, the term “years of occupational hazard exposure” refers to the length of time they have been exposed to non-noise occupational hazards, including dust, benzene, Pb, or high temperature. For the occupational noise-exposed group, “years of occupational hazard exposure” is essentially the same as “years of occupational noise exposure.”

### Pure tone audiometry test and classification of occupational noise exposure combined with BHFTA

A pure tone hearing threshold test is also required for workers who are about to engage in noise work and those who are engaged in noise work. To determine an individual’s hearing threshold levels, a PTA was performed by a trained audiologist using a verified pure tone audiometer. Workers underwent a PTA test after being away from occupational noise exposure for at least 48 h. The hearing thresholds of both ears were determined using the ascending pure tone method at frequencies of 0.5, 1, 2, 3, 4, and 6 kHz. For each frequency, trained audiologist would repeat tests until three responses occur at the same level out of a maximum of five ascents. Then, this level is defined as the hearing threshold level [25].

Based on the Standard for Occupational Health of the People’s Republic of China: Diagnosis of Occupational NIHL (GBZ 49–2014), workers whose BHFTA < 40 dB were defined as having normal BHFTA, while those with BHFTA ≥ 40 dB were defined as having elevated BHFTA, also known as BHFHL. To better describe the actual personnel exposure to occupational noise, we combined occupational noise exposure status and BHFTA level to further categorize the participants into 3 groups: “occupational noise non-exposed with BHFTA normal” group, which is the reference; “occupational noise exposed with BHFTA normal” groups and “occupational noise exposed with BHFTA elevated” groups.

### Measurement of blood pressure and definition of blood pressure and hypertension

SBP and DBP were measured by trained physicians using a verified electronic sphygmomanometer on the right arm at the heart level of participants who sat at rest for 5 min. Each participant was measured three times at 30-second intervals, and the final SBP and DBP were recorded as the average of the last two SBP and DBP readings, respectively. We defined possible SBP values as between 80 and 200 mmHg and DBP values as between 40 and 120 mmHg in our study. Values that fell outside this range were identified as false values and excluded.

According to 2018 Chinese guidelines for the management of hypertension [26], measured blood pressure data defined the following hypertension subtypes: hypertension (SBP ≥ 140 mmHg and/or a DBP ≥ 90 mmHg); stage 1 of hypertension (SBP 140—159 mm Hg and/or DBP 90—99 mm Hg), stage 2 of hypertension (SBP 160—179 mm Hg and/or DBP 100—109 mm Hg); stage 3 of hypertension (SBP ≥ 180 mm Hg and/or DBP ≥ 110 mm Hg); prehypertension (SBP 120—139 mm Hg and/or DBP 80—89 mm Hg); isolated systolic hypertension (ISH) (SBP ≥ 140 mm Hg and DBP < 90 mm Hg); normal blood pressure (SBP < 120 mm Hg and DBP <80 mm Hg).

### Statistical analysis

Descriptive analyses were performed to demonstrate the baseline characteristics of the included participants. Data were described as the means and SDs for normally distributed continuous variables and frequencies with percentages for categorical variables. Baseline characteristics were summarized by noise exposure status and compared between participants with and without occupational noise exposure using χ²-tests and analyses of variance, as appropriate.

Multiple linear regression models were performed to calculate the regression coefficient (β) and the corresponding standard error of the mean (SEM) to estimate the relationships of occupational noise exposure status, the combination of occupational noise exposure and BHFTA with blood pressure (SBP and DBP) in all participants. Unconditional logistic regression models were used to calculate crude and adjusted odds ratios (ORs) and the corresponding 95% confidence interval (95% CI) to examine the associations between occupational noise status, occupational noise exposure combined with BHFTA and hypertension. The fully adjusted model was controlled for sex (male, female), age, years of occupational hazard exposure, heat exposure (non-exposed, exposed), Pb exposure (non-exposed, exposed), benzene exposure (non-exposed, exposed), dust exposure (non-exposed, exposed), and industry type (non-manufacturing, manufacturing). Additionally, because sex and age have a critical influence on the development of hypertension, we conducted sex- and age-related subgroup analyses to further examine whether the observed associations of occupational noise exposure status, the combination of occupational noise exposure and BHFTA with hypertension would change. The interactions between occupational noise exposure and sex/age were examined using likelihood ratio test (LRT), with a comparison of the log likelihood of the two models with and without the interaction terms. Owing to the increased prevalence of hypertension with advancing age [27], we have categorized the ages into four groups: 18–29 years, 30–39 years, 40–49 years and 50–60 years. Furthermore, we estimated the attributable risk (AR) for the hypertension burden attributed to occupational exposure to noise [28], which indicate what proportion of the hypertension that could have been prevented in occupational noise-exposed workers. Five models were estimated: in model 1, we conducted the crude model without any adjustment. We used multivariate logistic regression to estimate the ORs adjusted for potential confounders using four models. Model 2 was adjusted for sex and age. Model 3 was adjusted for the same factors as model 2 and years of occupational hazard exposure. Model 4 was additionally adjusted for heat exposure, Pb exposure (non-exposed, exposed), benzene exposure (non-exposed, exposed), and dust exposure (non-exposed, exposed). Model 5 was adjusted for the same factor as model 4 and industries. A collinearity test using the variance inflation factor (VIF) (< 2, less than the cut-off value of VIF “10”) was used to determine the correlation between independent variables. No collinearity was detected among these covariates.

*P* values less than 0.05 were considered statistically significant. All *P* values are two-sided. Data were analysed with R version 4.2.3 and SAS 9.4.

### Role of the funding source

The funder of the study had no role in the study design, data collection, data analysis, data interpretation, or writing of the report.

## Results

### Characteristics of participants

A total of 715,135 participants (164,389 female and 550,746 male) were included in this study. The mean age of the participants was 36·1 ± 9·5 years. The prevalence of occupational noise exposure and hypertension was 97·8% and 13·7%, respectively. Blood pressure levels were normal for 32·9% of the participants, 53·4% had prehypertension, 9·5% had stage 1 hypertension, 3·3% had stage 2 hypertension, and 0·9% had stage 3 hypertension. The prevalence of hypertension was higher among occupational noise-exposed participants than among their counterparts (13·8% vs. 10·8%, *P* < 0:001), which was consistent within each classification of blood pressure. Table 1 presents the characteristics of the participants by occupational noise exposure status. Participants who were exposed to occupational noise were more likely to be younger, male, to have slightly higher levels of BHFTA, SBP, and DBP, and less likely to be exposed to high temperature, dust, and benzene but more likely to be exposed to Pb (all *P* < 0·001). Of all participants, 2·2% were Occupational noise non–exposed with BHFTA normal (15,666/715,135), 87.8% were Occupational noise exposed with BHFTA normal (627,822/715,135), and 10·0% were occupational noise exposed with BHFTA elevated (71,647/715,135). The BHFHL prevalence was 10·2% (71,647/699,469) in the occupational noise-exposed group.

**Table 1:** Basic characteristics of participants by occupational noise exposure

	Total (*N* = 715,135)	Occupational noise exposure		*P*
		Non–exposed (*N* = 15,666)	Exposed (*N* = 699,469)	
Age (years), mean (SD)	36.1 (9.5)	37.6 (9.9)	36.1 (9.5)	< 0.001
Years of occupational hazard exposure, mean (SD)	5.3 (4.7)	5.7 (4.5)	5.3 (4.8)	< 0.001
Sex, n (%)				< 0.001
Male	550,746 (77.0)	10,930 (69.8)	539,816 (77.2)	
Female	164,389 (23.0)	4,736 (30.2)	159,653 (22.8)	
Industry, n (%)				< 0.001
Manufacturing	616,748 (86.2)	14,010 (89.4)	602,738 (86.2)	
Other industries	98,387 (13.8)	1,656 (10.6)	96,731 (13.8)	
BHFTA, mean (SD)	24.6 (11.8)	21.1 (5.8)	24.6 (11.9)	< 0.001
BHFHL, n (%)				/
Yes	71,647(10.0)	-	71,647(10.2)	
No	643,488(90.0)	15,666(100.0)	627,822(89.8)	
SBP (mmHg), mean (SD)	124.2 (14.7)	123.0 (14.9)	124.3 (14.7)	< 0.001
DBP (mmHg), mean (SD)	78.9 (10.4)	77.9 (10.6)	78.9 (10.4)	< 0.001
Isolated systolic hypertension, n (%)				< 0.001
Yes	21,478 (8.4)	337 (5.6)	21,141 (8.4)	
No	235,311 (91.6)	5740 (94.5)	229,571 (91.6)	
Hypertension, n (%)				< 0.001
Yes	97,861 (13.7)	1691 (10.8)	96,170 (13.8)	
No	617,274 (86.3)	13,975 (89.2)	603,299 (86.2)	
Classification of blood pressure, n (%)				< 0.001
Normal	235,311 (32.9)	5740 (36.6)	229,571 (32.8)	
Prehypertension	381,963 (53.4)	8235 (52.6)	373,728 (53.4)	
Stage 1 hypertension	68,019 (9.5)	1176 (7.5)	66,843 (9.6)	
Stage 2 hypertension	23,287 (3.3)	379 (2.4)	22,908 (3.3)	
Stage 3 hypertension	6555 (0.9)	136 (0.9)	6419 (0.9)	
High temperature exposed, n (%)				< 0.001
Exposed	33,730 (4.7)	1654 (10.6)	32,076 (4.6)	
Non-exposed	681,405 (95.3)	14,012 (89.4)	667,393 (95.4)	
Dust exposed, n (%)				< 0.001
Exposed	306,974 (42.9)	10,125 (64.6)	296,849 (42.4)	
Non-exposed	408,161 (57.1)	5541 (35.4)	402,620 (57.6)	
Pb exposed, n (%)				< 0.001
Exposed	5923 (0.8)	80 (0.5)	5843 (0.8)	
Non-exposed	709,212 (99.2)	15,586 (99.5)	693,626 (99.2)	
Benzene exposed, n (%)				< 0.001
Exposed	52,153 (7.3)	4611 (29.4)	47,542 (6.8)	
Non-exposed	662,982 (92.7)	11,055 (70.6)	651,927 (93.2)	

-: Participants not exposed to occupational noise with BHFTA elevated have been excluded.

### Associations between occupational noise exposure and blood pressure

The mean levels (SD) of blood pressure (SBP and DBP) among participants who were occupational noise non-exposed with BHFTA normal, occupational noise exposed with BHFTA normal, and occupational noise exposed with BHFTA elevated were 123·0 (14.9)/77·9 (10.6), 123·9 (14·6)/78·7 (10·3) and 127·2 (15·6)/80·7 (10·8) mmHg, respectively. The unadjusted and adjusted linear regression models for occupational noise exposure and blood pressure (SBP and DBP) are shown in Table 2, which demonstrates that SBP and DBP were both significantly associated with the occupational exposure status and classification of occupational noise exposure combined with BHFTA in the crude and adjusted models (all *P* < 0·001). Regarding the status of occupational noise, the SBP and DBP of occupational noise-exposed workers were 1·63 (SEM = 0·12) mmHg and 1·32 (SEM = 0·08) mmHg higher than those of workers without occupational noise exposure (all *P* < 0·001). Regarding the classification of occupational noise exposure combined with BHFTA, workers in the occupational noise exposed with BHFTA elevated group had a higher SBP of 1·77 (SEM = 0·13) mmHg and DBP of 1·41 (SEM = 0·09) mmHg than those in the occupational noise non-exposed with BHFTA normal group (all *P* < 0·001). The estimates for the occupational noise exposed with BHFTA normal group were also significantly associated with higher SBP and DBP but weaker in magnitude (SBP: β = 1·61, SEM = 0·12, *P* < 0·001; DBP: β = 1·30, SEM = 0·08, *P* < 0·001; Table 2).

**Table 2:** Relationship between blood pressure and occupational exposure status, and classification of occupational noise exposure combined with BHFTA

	SBP, mmHg								DBP, mmHg							
	Model 1				Model 2				Model 1				Model 2			
	*β*	SEM	*P*		*β*	SEM	*P*		*β*	SEM	*P*		*β*	SEM	*P*	
**Occupational noise status**																
Non–exposed	ref				ref				ref				ref			
Exposed	1.30	0.12	< 0.001		1.63	0.12	< 0.001		0.95	0.08	< 0.001		1.32	0.08	< 0.001	
**Classification of occupational noise exposure combined with BHFTA**																
Occupational noise non–exposed with BHFTA normal	ref				ref				ref				ref			
Occupational noise exposed with BHFTA normal	0.97	0.12	< 0.001		1.61	0.12	< 0.001		0.74	0.08	< 0.001		1.30	0.08	< 0.001	
Occupational noise exposed with BHFTA elevated	4.20	0.13	< 0.001		1.77	0.13	< 0.001		2.77	0.09	< 0.001		1.41	0.09	< 0.001	

Model 1: Unadjusted

Model 2: Adjusted for sex, age, years of occupational hazard exposure, high temperature exposure (Non-exposed, exposed), benzene exposure (Non-exposed, exposed), Pb exposure (Non-exposed, exposed), dust exposure (Non-exposed, exposed), classification of industry (Manufacturing, Non–manufacturing)

SBP: systolic blood pressure

DBP: diastolic blood pressure

β: regression coefficient

SEM: standard error of the mean

### Associations between occupational noise exposure and hypertension

Compared with workers without occupational noise exposure, the risk of hypertension was 32% greater among those exposed to occupational noise (95% CI = 1·25–1·39) in the crude model. After adjustment for various covariates, the observed associations for hypertension remained statistically significant (OR = 1·50, 95% CI = 1·42–1·58). In comparison to the occupational noise non-exposed with BHFTA normal, the adjusted risks of hypertension were 1.48 (1·41–1·56) for the occupational noise exposed with BHFTA normal and 1.54 (1·46–1·63) for the occupational noise exposed with BHFTA elevated, respectively (Table 3). When examining other subtypes of blood pressure classifications, a similar association was found in ISH and prehypertension. (Table S1).

**Table 3:** Risk of prevalent hypertension associated with occupational exposure status and classification of occupational noise exposure combined with BHFTA

	Hypertension (≥ 140 mmHg/≥90 mmHg)								
	Model 1						Model 2		
	OR	95%CI	*P*			OR		95%CI	*P*
**Occupational noise status**									
Non–exposed	1					1			
Exposed	1.32	1.25,1.39	< 0.001			1.50		1.42,1.58	< 0.001
**Classification of occupational noise exposure combined with BHFTA**									
Occupational noise non–exposed with BHFTA normal	1					1			
Occupational noise exposed with BHFTA normal	1.25	1.19,1.31	< 0.001			1.48		1.41,1.56	< 0.001
Occupational noise exposed with BHFTA elevated	1.97	1.87,2.08	< 0.001			1.54		1.46,1.63	< 0.001

Model 1: Unadjusted

Model 2: Adjusted for sex, age, occupational hazard exposure years, high temperature exposure (Non-exposed, exposed), benzene exposure (Non-exposed, exposed), Pb exposure (Non-exposed, exposed), dust exposure (Non-exposed, exposed), classification of industry (Manufacturing, Non–manufacturing)

OR: odds ratio

CI: confidence interval

### Subgroup analysis

**Fig. 2:**
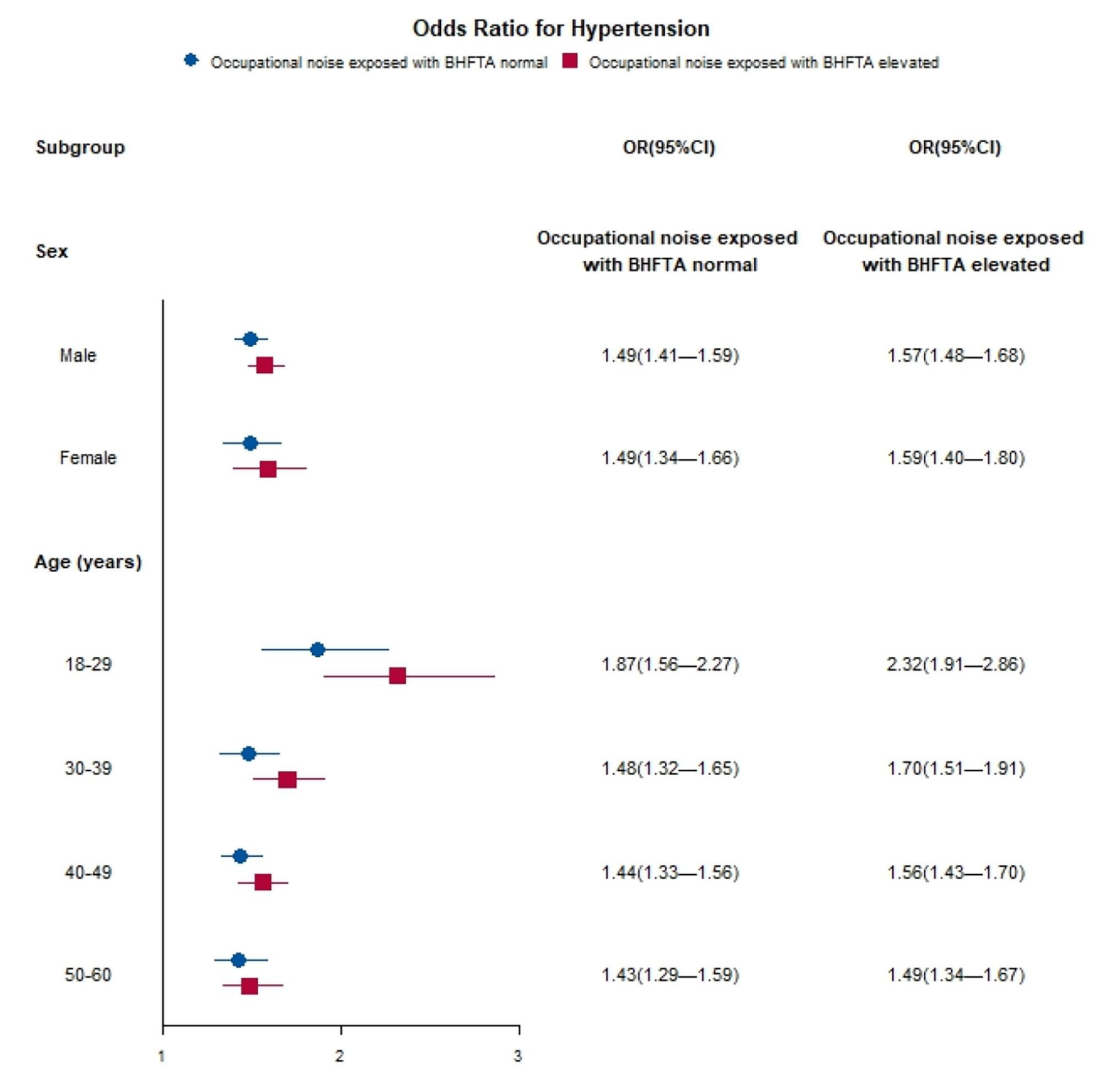
Subgroup analysis by sex and age for the associations between the prevalence of hypertension and classification of occupational noise exposure combined with BHFTA. Control group: Occupational noise non–exposed with BHFTA normal. OR odds ratio, CI confidence interval

In the subgroup analysis according to sex, there was a positive association between occupational noise exposure status, classification of occupational noise exposure combined with BHFTA and hypertension, ISH, and prehypertension in both males and females (Table 4, Table S2). There were no observed sex differences in the associations between noise exposure status and prevalence of hypertension subtypes (all *P* for interaction > 0.05). Compared to those not exposed to occupational noise, the adjusted OR (95% CI) for hypertension among occupational noise-exposed males was 1·51 (1·42–1·60) and among occupational noise-exposed females was 1·49 (1·34–1·67; Table 4). However, when examining the classification of occupational noise exposure combined with BHFTA, the associations with hypertension subtypes were stronger in the female group (all *P* for interaction < 0.005, Table 4; Fig. 2, Table S2, Fig S1).

**Table 4:** Subgroup analysis by sex for the associations between the prevalence of hypertension and occupational exposure status and classification of occupational noise exposure combined with BHFTA

Sex		Model 1				Model 2			*P* for interaction
		OR	95%CI	*P*		OR	95%CI	*P*	
	**Occupational noise status**								0.358
**Male**	Non–exposed	1				1			
	Exposed	1.29	1.22,1.37	< 0.001		1.51	1.42,1.60	< 0.001	
**Female**	Non–exposed	1				1			
	Exposed	1.28	1.15,1.41	< 0.001		1.49	1.34,1.67	< 0.001	
	**Classification of occupational noise exposure combined with BHFTA**								0.003
**Male**	Occupational noise non–exposed with BHFTA normal	1				1			
	Occupational noise exposed with BHFTA normal	1.22	1.15,1.29	< 0.001		1.49	1.41,1.59	< 0.001	
	Occupational noise exposed with BHFTA elevated	1.86	1.74,1.97	< 0.001		1.57	1.48,1.68	< 0.001	
**Female**	Occupational noise non–exposed with BHFTA normal	1				1			
	Occupational noise exposed with BHFTA normal	1.25	1.13,1.39	< 0.001		1.49	1.34,1.66	< 0.001	
	Occupational noise exposed with BHFTA elevated	1.87	1.66,2.11	< 0.001		1.59	1.40,1.80	< 0.001	

OR odds ratio, CI confidence interval

Model 1: Unadjusted

Model 2: Adjusted for age, years of occupational hazard exposure, high temperature exposure (Non-exposed, exposed), benzene exposure (Non-exposed, exposed), Pb exposure (Non-exposed, exposed), dust exposure (Non-exposed, exposed), classification of industry (Manufacturing, Non–manufacturing)

In the age-stratified subgroup analysis, we found consistently positive and significant associations between the occupational noise exposure status, the classification of occupational noise exposure combined with BHFTA, and the prevalence of hypertension and ISH across all age groups (all *P* < 0.001, Table 5; Fig. 2, Fig S1). However, there were no significant associations with respect to occupational noise exposure for prehypertension among workers older than 50 years (Table S3, Fig S2). Significant interactions between occupational noise status, classification of occupational noise exposure combined with BHFTA, and age in relation to hypertension risk were identified (all *P* for interaction < 0.001). The associations of occupational noise status, classification of occupational noise exposure combined with BHFTA and hypertension were most evident in the 18–29 age groups (occupational noise exposed: 1·90 [1·58–2·30]; occupational noise exposed with BHFTA normal: 1·87 [1·56–2·27], occupational noise exposed with BHFTA elevated: 2·32 [1·91–2·86]; Table 5; Fig. 2). A consistent pattern of interactions between occupational noise exposure and age with the risk of prehypertension was observed (Table S2, Fig S1). However, when examining the outcome of ISH, no significant interaction was found between occupational noise status and age (*P* for interaction = 0.345), but a significant interaction was found between the classification of occupational noise exposure combined with BHFTA and age (*P* for interaction = 0.035, Table S2, Fig S1).

**Table 5:** Subgroup analysis by age for the associations between the prevalence of hypertension and occupational exposure status and classification of occupational noise exposure combined with BHFTA

Age		Model 1				Model 2			*P* for interaction
		OR	95%CI	*P*		OR	95%CI	*P*	
	**Occupational noise status**								0.019
**18–29**	Non–exposed	1				1			
	Exposed	1.97	1.64,2.39	< 0.001		1.90	1.58,2.30	< 0.001	
**30–39**	Non–exposed	1				1			
	Exposed	1.57	1.41,1.75	< 0.001		1.50	1.34,1.68	< 0.001	
**40–49**	Non–exposed	1				1			
	Exposed	1.42	1.32,1.54	< 0.001		1.45	1.34,1.58	< 0.001	
**50–60**	Non–exposed	1				1			
	Exposed	1.44	1.30,1.60	< 0.001		1.44	1.30,1.60	< 0.001	
	**Classification of occupational noise exposure combined with BHFTA**								< 0.001
**18–29**	Occupational noise non–exposed with BHFTA normal	1				1			
	Occupational noise exposed with BHFTA normal	1.94	1.62,2.35	< 0.001		1.87	1.56,2.27	< 0.001	
	Occupational noise exposed with BHFTA elevated	2.57	2.11,3.15	< 0.001		2.32	1.91,2.86	< 0.001	
**30–39**	Occupational noise non–exposed with BHFTA normal	1				1			
	Occupational noise exposed with BHFTA normal	1.53	1.37,1.71	< 0.001		1.48	1.32,1.65	< 0.001	
	Occupational noise exposed with BHFTA elevated	1.94	1.73,2.19	< 0.001		1.70	1.51,1.91	< 0.001	
**40–49**	Occupational noise non–exposed with BHFTA normal	1				1			
	Occupational noise exposed with BHFTA normal	1.39	1.28,1.51	< 0.001		1.44	1.33,1.56	< 0.001	
	Occupational noise exposed with BHFTA elevated	1.62	1.49,1.76	< 0.001		1.56	1.43,1.70	< 0.001	
**50–60**	Occupational noise non–exposed with BHFTA normal	1				1			
	Occupational noise exposed with BHFTA normal	1.42	1.29,1.58	< 0.001		1.43	1.29,1.59	< 0.001	
	Occupational noise exposed with BHFTA elevated	1.51	1.36,1.69	< 0.001		1.49	1.34,1.67	< 0.001	

OR odds ratio, CI confidence interval

Model 1: Unadjusted

Model 2: Adjusted for sex, years of occupational hazard exposure, high temperature exposure (Non-exposed, exposed), benzene exposure (Non-exposed, exposed), Pb exposure (Non-exposed, exposed), dust exposure (Non-exposed, exposed), classification of industry (Manufacturing, Non–manufacturing)

### Attributable fraction of occupational noise with hypertension subtypes

Table 6 presents the AR% of occupational noise with hypertension subtypes in different models. The AR% of occupational noise exposure for hypertension were 21·12% in the crude model, ranging from 26·92% to 28·05% after adjusting for different covariates. Specifically, occupational noise exposure showed the highest AR% (33·70%—35·71%) for ISH but the lowest AR% (4·45%—5·70%) for prehypertension.

**Table 6:** AF% (95%CI) of occupational noise exposure with hypertension subtypes

	Hypertension	ISH	Prehypertension
**Model 1**	21.12% (17.60%,24.64%)	33.70% (26.87%,40.52%)	4.78% (3.47%,6.08%)
**Model 2**	26.92% (23.72%,30.12%)	35.68% (29.28%,42.08%)	4.45% (3.21%,5.76%)
**Model 3**	26.92% (23.72%,30.12%)	35.71% (29.31%,42.11%)	4.47% (3.19%,5.74%)
**Model 4**	27.41% (24.22%,30.61%)	33.93% (27.34%,40.51%)	5.39% (4.10%,6.68%)
**Model 5**	28.05% (24.87%,31.22%)	35.13% (28.64%,41.62%)	5.70% (4.41%,7.00%)

Control group: Occupational noise non-exposed

Model 1: Unadjusted

Model 2: Adjusted for sex and age

Model 3: Adjusted for sex, age and years of occupational hazard exposure

Model 4: Adjusted for sex, age, years of occupational hazard exposure, high temperature exposure (Non-exposed, exposed), benzene exposure (Non-exposed, exposed), Pb exposure (Non-exposed, exposed) and dust exposure (Non-exposed, exposed)

Model 5: Adjusted for sex, age, years of occupational hazard exposure, high temperature exposure (Non-exposed, exposed), benzene exposure (Non-exposed, exposed), Pb exposure (Non-exposed, exposed), dust exposure (Non-exposed, exposed) and classification of industry (Manufacturing, Non–manufacturing)

## Discussion

In this large-scale occupational population-based study, we investigated the associations of occupational noise exposure with blood pressure and hypertension in a sample of 715,135 participants with occupational hazards exposure. Occupational noise exposure was significantly associated with an increase in SBP, DBP levels, and the prevalence of hypertension, ISH, and prehypertension. These associations remained significant after adjusting for various covariates and in different sex or age groups. Our findings also pointed toward an increased risk of hypertension associated with occupational noise exposure, particularly pronounced in individuals with elevated BHFTA. Participants in occupational noise exposed with BHFTA elevated group had the most profound impact on having hypertension. Occupational noise was responsible for hypertension with a AR of 28·05%, implying that more than one-fourth of hypertension cases could be prevented by avoiding occupational noise exposure.

Our study results indicated that occupational noise exposure was significantly associated with an increase in the prevalence of hypertension. The findings were consistent with some previous articles. Lin et al. [29] suggested that exposure to noise levels between 82 and 106 dB(A) for 3–17 years may increase the risk of hypertension with a nonlinear exposure response pattern. Chen et al. [30] also found that occupational noise exposure was associated with higher levels of SBP, DBP, and the risk of hypertension in 1,390 occupational noise-exposed workers and 1,399 frequency matched non noise-exposed subjects. Some other epidemiological studies did not support the positive association of occupational noise and hypertension. A 7-years prospective cohort study showed no increased risk of hypertension with exposure to noise in the lower half of the 80–90 dB(A) range [17]. Similarly, Tessier-Sherman et al. [31] found that the adjusted hazard ratio (HR) of incident hypertension did not significantly differ between groups by cumulative continuous or categorized noise exposure metrics among specialty metal manufacturing workers in a 6-years cohort study. By comparison, these published studies agreed in principle but not in detail. These inconsistent results might be due to the different populations, sample sizes, study designs, study regions, definitions of occupational noise exposure, and confounding variables across different studies.

To the extent that previous studies have focused on the strength of the association between occupational noise exposure and hypertension, however, they have not reported the AF of occupational noise for hypertension. Since the prevalence of occupational noise exposure is high, the fraction of hypertension that could be attributable to noise exposure can be substantial [19]. We calculated that in the fully adjusted model, the AR% of hypertension due to occupational noise exposure stood at 28.05%, indicating that eliminating exposure to occupational noise could potentially prevent over a quarter of hypertension cases. In the future, more research is needed to elucidate the burden of occupational noise exposure on the risk of hypertension in different countries to confirm our results.

We have found that SBP and DBP mean levels are significantly higher in the presence of occupational noise even if it does not reach abnormal limits. A meta-analysis conducted by Tomei et al. [10] with 15 studies showed a statistically significant increase in SBP and DBP in high-exposure workers compared to low-exposure and immediate-exposure workers. However, Gan et al. [32] did not find significant alterations in the levels of SBP, DBP or the prevalence of self-reported hypertension or general hypertension for self-reported noise-exposed participants. The inconsistency might result from the assessment of occupational noise exposure. In our study, occupational noise exposure had a relatively higher OR of ISH than hypertension. This might suggest that SBP might be used as a screening tool to identify initial alterations in the cardiovascular system in workers exposed to occupational noise.

Previous studies have only examined the relationship between BHFHL and hypertension in noise-exposed workers [21, 33] and did not investigate the relationship between the occupational noise exposure combined with BHFTA and hypertension by taking none noise-exposed workers as a reference. Thus, to better reflect the actual personal exposure to occupational, we combined the status of occupational noise exposure with BHFTA level as an indicator to reflect the cumulative exposure of occupational noise. Our findings indicate that individuals exposed to occupational noise with BHFTA normal face a significantly higher risk of hypertension compared to those with BHFTA normal and no occupational noise exposure. Furthermore, individuals exposed to occupational noise with BHFTA elevated experience an even greater elevation in risk. Similar patterns were observed when the relationship was evaluated for ISH and hypertension.

The biological mechanism underlying noise-induced hypertension is not fully understood. Most of the proposed pathways are from environmental sources of noise exposure [34–38]. It has been proposed that noise may perturb the autonomic nervous system and/or overactive sympathetic nervous system, directly or indirectly modulating the sympathetic adrenal-medullary (SAM) axis, the hypothalamic‒pituitary‒adrenal (HPA) axis, and the renin-angiotensin system (RAS) to increase the secretion of catecholamines (norepinephrine, epinephrine, and dopamine), cortisol and angiotensin II, which could result in the elevation of blood pressure [34, 39, 40]. In addition, noise could trigger a series of systematic inflammation and oxidative stress responses. These responses may further cause endothelial dysfunction, eventually leading to elevated blood pressure [35, 41].

Subgroup analysis indicated that positive associations between occupational noise exposure and hypertension remained significant in the subgroup of different sex and age groups, suggesting that the observed associations were stable and would not be modified by sex or age, which could verify the result of occupational noise and hypertension in our study. Moreover, sex differences in the associations were found, with the point estimates being slightly higher for males than for females, which could also be observed in other studies [21, 33, 42]. However, the Dongfeng-Tongji cohort study found an association with a 16% increase in hypertension risk only among males, not among females [33]. The sex difference might be due to the differences in auditory sensitivity and pathogenesis of cardiovascular diseases. It has been reported that men are more susceptible than women to the relationship between noise and cardiovascular diseases [43]. Additionally, it could be related to differences in jobs between males and females. Compared to male workers, female workers may experience a lower noise working environment. However, some other studies found opposite results and argued that women are probably more susceptible to the cardiometabolic effects of noise [44]. Furthermore, an age difference in associations was also found, with the effects of noise exposure intensity on hypertension attenuated with increasing age. It is well known that age is a key factor contributing to the development of hypertension. In a subgroup analysis by age, the OR between occupational noise exposure and hypertension was highest in the younger group (18–24 years), suggesting that younger people are probably more susceptible to the cardiometabolic effects of noise than older people.

Compared with previous studies that also investigated the association between occupational noise exposure and hypertension, the major strength of our study is that we combined occupational noise exposure and BHFTA to more accurately estimate the true intensity of occupational noise exposure for each individual and to further explore the relationships between occupational noise and different hypertension subtypes and blood pressure levels, which provide better evidence on the reliability of observed associations of occupational noise exposure and hypertension. Furthermore, to our knowledge, this is by far the largest study of associations between occupational noise exposure and hypertension, which may allow for a more robust analysis with better generalizability.

Despite its significant contributions, this study has several limitations. First, there are no direct measurements of noise exposure. Because the Key Occupational Diseases Surveillance Project uses a qualitative variable to define whether a person is exposed to occupational noise, it does not reflect various quantitative characteristics of noise, such as loudness, kurtosis, and frequency. Further studies using comprehensive occupational noise exposure data at working sites and personnel are needed. Second, some important information was not collected, such as the usage of hearing protection equipment, exposure to environmental noise, BMI, lifestyle characteristics, etc., which were also considered potential confounders of hearing loss and hypertension. Therefore, in future studies, we will conduct a large longitudinal study based our projects by collecting the above missing data to verify whether there is a causal relationship between occupational noise and hypertension.

## Conclusion

In summary, our study further revealed that occupational exposure to noise was positively associated with the prevalence of hypertension and blood pressure levels in a large occupational population-based study. Our results indicated a significantly increased risk of hypertension even in individuals with normal BHFTA exposed to occupational noise, with a further elevated risk observed in those with elevated BHFTA. A significantly increased risk of hypertension was found even in individuals with normal BHFTA exposed to occupational noise, with a further elevated risk observed in those with elevated BHFTA. Sex-related and age-related subgroup analyses showed positive significant associations between occupational noise exposure and hypertension remained statistically significant across all subgroups. The associations of occupational noise status, classification of occupational noise exposure combined with BHFTA and hypertension were most pronounced in the 18–29 age groups.

Given the rapidly industrialized society, widespread noise exposure, and heavy burden of hypertension, our findings provide epidemiological evidence for key groups associated with occupational noise exposure and hypertension, and more than one-fourth of hypertension cases would have been prevented by avoiding occupational noise exposure. Excess noise exposure in the workplace is an important occupational health issue and deserves special attention.

### Electronic supplementary material

Below is the link to the electronic supplementary material.

Supplementary Material 1

## Data Availability

The datasets and analysis will be available upon request. The researchers retain ownership of their data. Any requests for access to data should be made directly to corresponding authors.

## Abbreviations

95% CI: 95% Confidence Interval
AF: Attributable fraction
AR: Attributable Risk
PTA: Pure tone audiometry
SBP: Systolic blood pressure
DBP: Diastolic blood pressure
SEM: Standard error of the mean
BHFTA: Binaural high frequency (3, 4, and 6 kHz) threshold on average
BHFHL: Bilateral high-frequency average hearing loss
