# Supplementary material for: Occupational noise and hypertension in Southern Chinese workers: a large occupational population-based study
Source: BMC Public Health. 2024 Feb 21;24:541. doi: 10.1186/s12889-024-18040-9 (PMC10882732; doi:10.1186/s12889-024-18040-9)
Supplement: Supplementary file 1 — Supplementary Material 1 [file 12889_2024_18040_MOESM1_ESM.docx]

Table S1 Risk of prevalent ISH and prehypertension associated with occupational exposure status and classification of occupational noise exposure combined with BHFTA

|  | Isolated systolic hypertension (≥140/<90 mmHg) | | | | | | |  | Prehypertension (120~139 mmHg /80~89 mmHg) | | | | | | |
| --- | --- | --- | --- | --- | --- | --- | --- | --- | --- | --- | --- | --- | --- | --- | --- |
|  | Model 1 | | |  | Model 2 | | |  | Model 1 | | |  | Model 2 | | |
|  | OR | 95%CI | *P* |  | OR | 95%CI | *P* |  | OR | 95%CI | *P* |  | OR | 95%CI | *P* |
| **Occupational noise status** |  |  |  |  |  |  |  |  |  |  |  |  |  |  |  |
| Non–exposed | 1 |  |  |  | 1 |  |  |  | 1 |  |  |  | 1 |  |  |
| Exposed | 1.57 | 1.40,1.75 | <0.001 |  | 1.66 | 1.48,1.86 | <0.001 |  | 1.14 | 1.10,1.17 | <0.001 |  | 1.17 | 1.13,1.21 | <0.001 |
| **Classification of occupational noise exposure combined with BHFTA** |  |  |  |  |  |  |  |  |  |  |  |  |  |  |  |
| Occupational noise non–exposed  with BHFTA normal | 1 |  |  |  | 1 |  |  |  | 1 |  |  |  | 1 |  |  |
| Occupational noise exposed  with BHFTA normal | 1.47 | 1.32,1.65 | <0.001 |  | 1.65 | 1.47,1.85 | <0.001 |  | 1.11 | 1.07,1.15 | <0.001 |  | 1.17 | 1.13,1.21 | <0.001 |
| Occupational noise exposed  with BHFTA elevated | 2.61 | 2.32,2.93 | <0.001 |  | 1.70 | 1.51,1.92 | <0.001 |  | 1.42 | 1.36,1.47 | <0.001 |  | 1.15 | 1.11,1.20 | <0.001 |

Model1: Unadjusted

Model2: Adjusted gender, age, years of occupational hazard exposure, high temperature exposure (Non-exposed, exposed), benzene exposure (Non-exposed, exposed), Pb exposure (Non-exposed, exposed), dust exposure (Non-exposed, exposed), classification of industry (Manufacturing, Non–manufacturing)

OR odds ratio, CI confidence interval

Table S2 Subgroup analysis by sex for the prevalence of ISH and prehypertension associated with occupational exposure status and classification of occupational noise exposure combined with BHFTA

| **Sex** |  | **Isolated systolic hypertension (≥140/<90 mmHg)** | | | | | | | |  | **Prehypertension (120~139 mmHg /80~89 mmHg)** | | | | | | | |
| --- | --- | --- | --- | --- | --- | --- | --- | --- | --- | --- | --- | --- | --- | --- | --- | --- | --- | --- |
|  |  | **Model 1** | | |  | **Model 2** | | | ***P* for**  **interaction** |  | **Model 1** | | |  | **Model 2** | | | ***P* for**  **interaction** |
|  |  | **OR** | **95%CI** | ***P*** |  | **OR** | **95%CI** | ***P*** |  |  | **OR** | **95%CI** | ***P*** |  | **OR** | **95%CI** | ***P*** |  |
|  | **Occupational noise status** |  |  |  |  |  |  |  | 0.808 |  |  |  |  |  |  |  |  | 0.962 |
| **Male** | Non–exposed | 1 |  |  |  | 1 |  |  |  |  | 1 |  |  |  | 1 |  |  |  |
|  | Exposed | 1.49 | 1.31,1.70 | <0.001 |  | 1.62 | 1.42,1.86 | <0.001 |  |  | 1.08 | 1.04,1.13 | <0.001 |  | 1.15 | 1.11,1.40 | <0.001 |  |
| **Female** | Non–exposed | 1 |  |  |  | 1 |  |  |  |  | 1 |  |  |  | 1 |  |  |  |
|  | Exposed | 1.56 | 1.27,1.93 | <0.001 |  | 1.87 | 1.50,2.35 | <0.001 |  |  | 1.09 | 1.03,1.16 | <0.001 |  | 1.21 | 1.14,1.29 | <0.001 |  |
|  | **Classification of occupational noise exposure combined with BHFTA** |  |  |  |  |  |  |  | 0.002 |  |  |  |  |  |  |  |  | <0.001 |
| **Male** | Occupational noise non–exposed  with BHFTA normal | 1 |  |  |  | 1 |  |  |  |  | 1 |  |  |  | 1 |  |  |  |
|  | Occupational noise exposed  with BHFTA normal | 1.40 | 1.23,1.60 | <0.001 |  | 1.61 | 1.41,1.84 | <0.001 |  |  | 1.06 | 1.02,1.11 | 0.002 |  | 1.15 | 1.10,1.20 | <0.001 |  |
|  | Occupational noise exposed  with BHFTA elevated | 2.29 | 2.00,2.63 | <0.001 |  | 1.72 | 1.50,1.98 | <0.001 |  |  | 1.22 | 1.17,1.28 | <0.001 |  | 1.16 | 1.10,1.21 | <0.001 |  |
| **Female** | Occupational noise non–exposed  with BHFTA normal | 1 |  |  |  | 1 |  |  |  |  | 1 |  |  |  | 1 |  |  |  |
|  | Occupational noise exposed  with BHFTA normal | 1.52 | 1.24,1.89 | <0.001 |  | 1.86 | 1.50,2.34 | <0.001 |  |  | 1.08 | 1.02,1.15 | <0.001 |  | 1.21 | 1.13,1.29 | <0.001 |  |
|  | Occupational noise exposed  with BHFTA elevated | 2.71 | 2.13,3.45 | <0.001 |  | 1.96 | 1.52,2.54 | <0.001 |  |  | 1.37 | 1.26,1.48 | <0.001 |  | 1.26 | 1.16,1.37 | <0.001 |  |

OR odds ratio, CI confidence interval

Model1: Unadjusted

Model2: Adjusted age, years of occupational hazard exposure, high temperature exposure (Non-exposed, exposed), benzene exposure (Non-exposed, exposed), Pb exposure (Non-exposed, exposed), dust exposure (Non-exposed, exposed), classification of industry (Manufacturing, Non–manufacturing)

Table S3 Subgroup analysis by age for the prevalence of ISH and prehypertension associated with occupational exposure status and classification of occupational noise exposure combined with BHFTA

| **Age** |  | **Isolated systolic hypertension (≥140/<90 mmHg)** | | | | | | | |  | **Prehypertension (120~139 mmHg /80~89 mmHg)** | | | | | | | |
| --- | --- | --- | --- | --- | --- | --- | --- | --- | --- | --- | --- | --- | --- | --- | --- | --- | --- | --- |
|  |  | **Model 1** | | |  | **Model 2** | | | ***P* for**  **interaction** |  | **Model 1** | | |  | **Model 2** | | | ***P* for**  **interaction** |
|  |  | **OR** | **95%CI** | ***P*** |  | **OR** | **95%CI** | ***P*** |  |  | **OR** | **95%CI** | ***P*** |  | **OR** | **95%CI** | ***P*** |  |
|  | **Occupational noise status** |  |  |  |  |  |  |  | 0.345 |  |  |  |  |  |  |  |  | <0.001 |
| **18-29** | Non–exposed | 1 |  |  |  | 1 |  |  |  |  | 1 |  |  |  | 1 |  |  |  |
|  | Exposed | 2.16 | 1.58,3.06 | <0.001 |  | 1.87 | 1.36,2.66 | <0.001 |  |  | 1.37 | 1.28,1.46 | <0.001 |  | 1.32 | 1.23,1.41 | <0.001 |  |
| **30-39** | Non–exposed | 1 |  |  |  | 1 |  |  |  |  | 1 |  |  |  | 1 |  |  |  |
|  | Exposed | 1.85 | 1.45,2.42 | <0.001 |  | 1.69 | 1.31,2.21 | <0.001 |  |  | 1.19 | 1.13,1.27 | <0.001 |  | 1.17 | 1.10,1.24 | <0.001 |  |
| **40-49** | Non–exposed | 1 |  |  |  | 1 |  |  |  |  | 1 |  |  |  | 1 |  |  |  |
|  | Exposed | 1.74 | 1.46,2.10 | <0.001 |  | 1.75 | 1.46,2.12 | <0.001 |  |  | 1.11 | 1.04,1.19 | 0.001 |  | 1.13 | 1.05,1.20 | <0.001 |  |
| **50-60** | Non–exposed | 1 |  |  |  | 1 |  |  |  |  | 1 |  |  |  | 1 |  |  |  |
|  | Exposed | 1.56 | 1.26,1.94 | <0.001 |  | 1.58 | 1.27,1.97 | <0.001 |  |  | 0.93 | 0.82,1.04 | 0.202 |  | 0.94 | 0.83,1.06 | 0.332 |  |
|  | **Classification of occupational noise exposure combined with BHFTA** |  |  |  |  |  |  |  | 0.035 |  |  |  |  |  |  |  |  | <0.001 |
| **18-29** | Occupational noise non–exposed  with BHFTA normal | 1 |  |  |  | 1 |  |  |  |  | 1 |  |  |  | 1 |  |  |  |
|  | Occupational noise exposed  with BHFTA normal | 2.12 | 1.55,3.01 | <0.001 |  | 1.85 | 1.35,2.63 | <0.001 |  |  | 1.37 | 1.28,1.46 | <0.001 |  | 1.32 | 1.23,1.41 | <0.001 |  |
|  | Occupational noise exposed  with BHFTA elevated | 2.89 | 2.05,4.20 | <0.001 |  | 2.18 | 1.54,3.18 | <0.001 |  |  | 1.47 | 1.36,1.58 | <0.001 |  | 1.29 | 1.19,1.40 | <0.001 |  |
| **30-39** | Occupational noise non–exposed  with BHFTA normal | 1 |  |  |  | 1 |  |  |  |  | 1 |  |  |  | 1 |  |  |  |
|  | Occupational noise exposed  with BHFTA normal | 1.81 | 1.41,2.36 | <0.001 |  | 1.67 | 1.30,2.19 | <0.001 |  |  | 1.18 | 1.11,1.25 | <0.001 |  | 1.17 | 1.10,1.24 | <0.001 |  |
|  | Occupational noise exposed  with BHFTA elevated | 2.40 | 1.85,3.17 | <0.001 |  | 1.82 | 1.39,2.41 | <0.001 |  |  | 1.38 | 1.29,1.47 | <0.001 |  | 1.19 | 1.11,1.27 | <0.001 |  |
| **40-49** | Occupational noise non–exposed  with BHFTA normal | 1 |  |  |  | 1 |  |  |  |  | 1 |  |  |  | 1 |  |  |  |
|  | Occupational noise exposed  with BHFTA normal | 1.70 | 1.42,2.05 | <0.001 |  | 1.72 | 1.44,2.08 | <0.001 |  |  | 1.09 | 1.02,1.16 | 0.010 |  | 1.12 | 1.05,1.20 | 0.001 |  |
|  | Occupational noise exposed  with BHFTA elevated | 2.04 | 1.70,2.48 | <0.001 |  | 1.96 | 1.63,2.39 | <0.001 |  |  | 1.27 | 1.19,1.36 | <0.001 |  | 1.17 | 1.09,1.25 | <0.001 |  |
| **50-60** | Occupational noise non–exposed  with BHFTA normal | 1 |  |  |  | 1 |  |  |  |  | 1 |  |  |  | 1 |  |  |  |
|  | Occupational noise exposed  with BHFTA normal | 1.53 | 1.24,1.90 | <0.001 |  | 1.55 | 1.25,1.93 | <0.001 |  |  | 0.91 | 0.81,1.03 | 0.128 |  | 0.94 | 0.83,1.05 | 0.281 |  |
|  | Occupational noise exposed  with BHFTA elevated | 1.70 | 1.36,2.14 | <0.001 |  | 1.74 | 1.39,2.19 | <0.001 |  |  | 0.99 | 0.87,1.12 | 0.852 |  | 0.97 | 0.86,1.11 | 0.693 |  |

OR odds ratio, CI confidence interval

Model1: Unadjusted

Model2: Adjusted gender, years of occupational hazard exposure, high temperature exposure (Non-exposed, exposed), benzene exposure (Non-exposed, exposed), Pb exposure (Non-exposed, exposed), dust exposure (Non-exposed, exposed), classification of industry (Manufacturing, Non–manufacturing)

a.
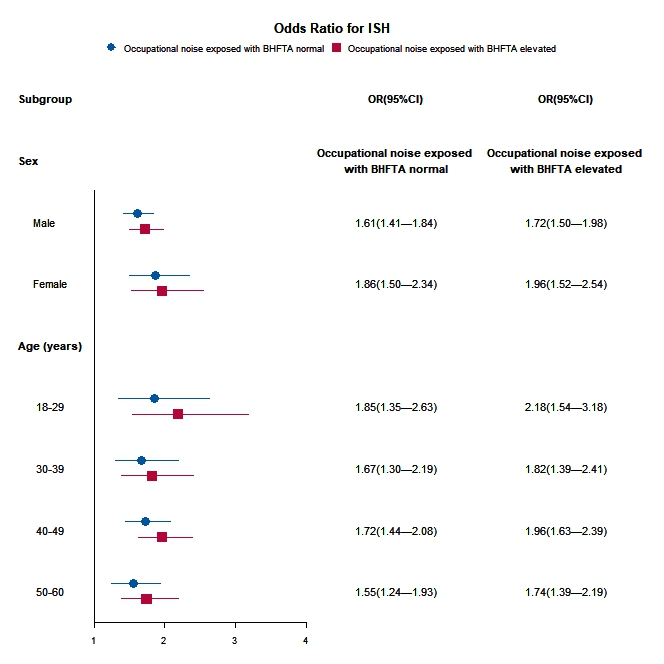
 b.
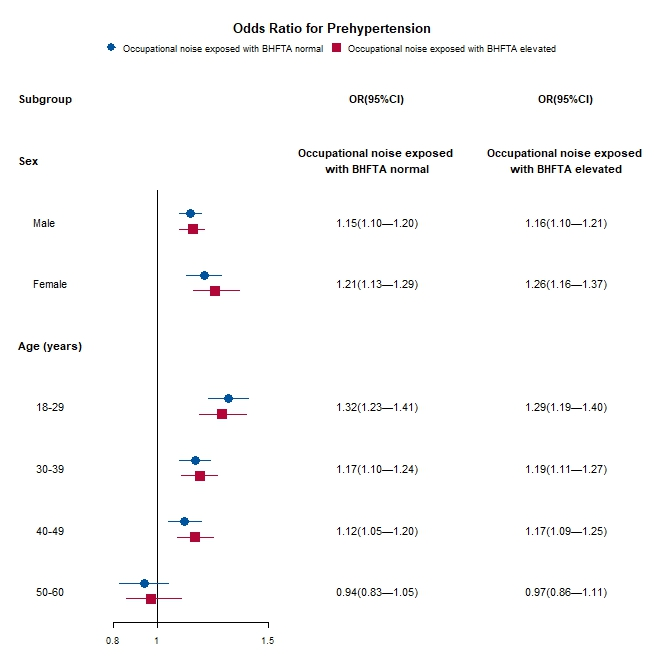


Figure S1 Subgroup analysis by sex and age for the prevalence of ISH and prehypertension associated with the classification of occupational noise exposure combined with BHFTA

Control group: Occupational noise non–exposed with BHFTA normal

OR odds ratio, CI confidence interval
